# Dose-response effects of estrogenic mycotoxins (zearalenone, alpha- and beta-zearalenol) on motility, hyperactivation and the acrosome reaction of stallion sperm

**DOI:** 10.1186/1477-7827-9-134

**Published:** 2011-10-05

**Authors:** Angela Filannino, Tom AE Stout, Bart M Gadella, Edita Sostaric, Flavia Pizzi, Ben Colenbrander, Maria Elena Dell'Aquila, Fiorenza Minervini

**Affiliations:** 1 Department of Animal Production, University Aldo Moro of Bari, Italy; 2 Department of Equine Sciences, Faculty of Veterinary Medicine, University of Utrecht, Utrecht, The Netherlands; 3 Institute of Agricultural Biology and Biotechnology (IBBA) National Research Council (CNR) Milano, Italy; 4 Institute of Sciences of Food Production (ISPA), National Research Council (CNR) Bari, Italy

## Abstract

**Background:**

The aim of this study was to investigate the *in vitro *effects of the Fusarium fungus-derived mycotoxin, zearalenone and its derivatives alpha-zearalenol and beta-zearalenol on motility parameters and the acrosome reaction of stallion sperm. Since the toxic effects of zearalenone and its derivatives are thought to result from their structural similarity to 17beta-estradiol, 17beta-estradiol was used as a positive control for 'estrogen-like' effects.

**Methods:**

Stallion spermatozoa were exposed *in vitro *to zearalenone, alpha-zearalenol, beta-zearalenol or 17beta-estradiol at concentrations ranging from 1 pM - 0.1 mM. After 2 hours exposure, motility parameters were evaluated by computer-assisted analysis, and acrosome integrity was examined by flow cytometry after staining with fluoroscein-conjugated peanut agglutinin.

**Results:**

Mycotoxins affected sperm parameters only at the highest concentration tested (0.1 mM) after 2 hours exposure. In this respect, all of the compounds reduced the average path velocity, but only alpha-zearalenol reduced percentages of motile and progressively motile sperm. Induction of motility patterns consistent with hyperactivation was stimulated according to the following rank of potency: alpha-zearalenol >17beta-estradiol > zearalenone = beta-zearalenol. The hyperactivity-associated changes observed included reductions in straight-line velocity and linearity of movement, and an increase in the amplitude of lateral head displacement, while curvilinear velocity was unchanged. In addition, whereas alpha- and beta- zearalenol increased the percentages of live acrosome-reacted sperm, zearalenone and 17beta-estradiol had no apparent effect on acrosome status. In short, alpha-zearalenol inhibited normal sperm motility, but stimulated hyperactive motility in the remaining motile cells and simultaneously induced the acrosome reaction. Beta-zearalenol induced the acrosome reaction without altering motility. Conversely, zearalenone and 17beta-estradiol did not induce the acrosome reaction but induced hyperactive motility albeit to a different extent.

**Conclusions:**

Apparently, the mycotoxin zearalenone has 17beta-estradiol-like estrogenic activity that enables it to induce hyperactivated motility of equine sperm cells, whereas the zearalenol derivatives induce premature completion of the acrosome reaction and thereby adversely affect stallion sperm physiology. The alpha form of zearalenol still possessed the estrogenic ability to induce hyperactivated motility, whereas its beta stereo-isomere had lost this property.

## Background

Zearalenone (ZEA) is an estrogenic mycotoxin biosynthesized by a variety of fungal species of the genus *Fusarium*, which are commonly found in the soil in temperate and warm countries and are frequent contaminants of cereal crops worldwide [1]. The toxic effects of Zearalenone and its metabolites have been ascribed primarily to its chemical structure that resembles that of naturally occurring estrogens [2]. Zearalenone is rapidly absorbed following oral intake and, during subsequent metabolism mainly in the liver and intestine, it is transformed into α- and β-zearalenol (α- and β-ZOL), α- and β-zearalanol (α- and β-ZAL) and zearalanone (ZAN), all of which are subsequently conjugated to glucuronic acid [2]. A variety of other tissues, including the kidney, testis, prostate, hypothalamus and ovary, also contain the major enzymes (3α- and 3β-hydroxysteroid dehydrogenase) able to metabolize mycotoxins [3]. Elimination of ZEA and its derivatives occurs mainly via the urine [4]. Zearalenone and its derivatives are thought to exert toxicity both by competitive binding to specific estrogen receptors and by modification of steroid metabolism [5].

In the horse, urinary concentrations of ZEA and its derivatives after natural exposure have been reported to range from 0.5 nM to 6.8 μM [6,7]. In pigs, levels of ZEA and α-ZOL ranging from 8.2 nM to 1 μM and from 0.13 μM to 0.87 μM have been detected in blood plasma and urine, respectively, during experimental intoxication [8-12]; i.e., the concentrations were found to be higher in urine than blood. In follicular fluid from naturally exposed pigs and cattle, ZEA and its derivatives have been detected at levels ranging from 30 pM to 0.2 nM and from 10 to 60 pM, respectively [13,14]. However, there are no reports of the concentrations of ZEA and its derivatives in body fluids of horses during experimental intoxication, and no data is available for the concentrations of mycotoxins in seminal plasma.

In domestic animal species, putative toxic effects of ZEA on reproductive performance have been investigated primarily in female animals, particularly cattle and pigs, whereas relatively few studies have examined the influence of ZEA on male reproductive function [15]. In adult rams, however, daily intake of 12 mg ZEA for 8 weeks during the breeding season affected sperm production and the percentages of motile and morphologically normal sperm [16]. To our knowledge, there are no reports of the effects of ZEA and its derivatives on semen quality or fertility in bulls. In pigs, *in vitro *studies have reported toxic effects of ZEA and its derivatives on sperm parameters thought to be important for fertility [17-19]; in particular, ZEA and its derivatives reduced the percentages of viable and progressively motile sperm [17,19] and the percentages of sperm able to undergo the acrosome reaction (AR) [17] or which contained stable chromatin [18,19]. All of these changes would be expected to reduce fertilizing capacity, and may in part explain the observed reductions in farrowing rate and total number of piglets born in ZEA affected pig herds [17].

In contrast to pigs and ruminants, horses are thought to be relatively insensitive to the effects of ZEA on reproductive function. Aurich et al. [20] reported that the concentrations of the toxin found in naturally contaminated oats had no significant effect on the release of reproductive hormones, cycle length, or uterine histology in mares. On the other hand, we previously reported toxic effects at the level of sperm chromatin stability in equine sperm exposed to low concentrations (up to 0.025 pM) of ZEA and its derivatives (α-ZOL, β-ZOL, α-ZAL, β -ZAL and ZAN) *in vitro*, suggesting that a genotoxic effect was being stimulated by a non-genomic mechanism [6].

A significant body of experimental evidence has also been accumulated to suggest that steroid hormones can influence reproductive function via plasma membrane receptors and, thereby, exert their effects via non-genomic pathways. For example, Giammarino et al. [21] reported that ZEA, α-ZOL and β-ZOL rapidly enhanced spontaneous contraction of isolated lamb uterine smooth muscle in a fashion similar to 17β-estradiol (E_2_); this supported the existence of non-genomic pathways for the actions of sex steroids because the effect was too rapid to be explained by transcription of RNA and subsequent protein synthesis. The concept that steroid hormones may act via pathways other than the classical genomic route has attracted particular attention for sperm since the latter are transcriptionally inactive. Indeed, the demonstration that sex steroids can affect sperm function has helped to strengthen the idea that steroids can act via non-genomic pathways [22]. To date, however, studies on the effects of estrogens on sperm function are limited in number, and both species-specific differences and apparently contradictory results have been reported. Baldi et al. [22] performed an exhaustive review of recent investigations into non-genomic activation of spermatozoa by steroid hormones; they concluded that *in vitro *treatment of human sperm with E_2 _induces a small rise in intracellular calcium concentrations that, ultimately, inhibits the progesterone-induced AR. However, E_2 _did not appear to directly affect the AR. These effects of E_2 _on human sperm were recently confirmed by Vigil et al. [23]. By contrast, Francavilla et al. [24] reported that pre-incubation with E_2 _did not alter the ability of human sperm to fuse with an oocyte, nor did it interfere with the ability of progesterone to enhance this process.

In the stallion, E_2 _levels ranging from 0.1 - 0.4 nM in blood plasma [25,26] and from 10 pM to 2 nM in seminal plasma [25-27] have been reported. In the follicular fluid of mares, E_2 _concentrations are generally higher and more variable, at between 20 nM and 11 μM [28-32].

To our knowledge, there are no reports of the effects of ZEA or its derivatives on sperm motility and the AR in the horse. The aim of the present study was to determine whether ZEA and its derivatives, α-ZOL and β-ZOL, exert dose-dependent effects on equine sperm motility, including hyperactive motility, as assessed by computer assisted sperm analysis (CASA), and/or on the AR, as assessed by fluoroscein isothiocyanate conjugated peanut agglutinin (FITC-PNA) staining and flow cytometry. To further clarify whether the effects of mycotoxins on stallion sperm could be considered to be estrogen-like, the effects of *in vitro *exposure of sperm to E_2 _were also investigated.

## Methods

### Materials

The following key reagents were used in these studies; propidium iodide (PI, Molecular Probes, Eugene, OR), fluorescein isothiocyanate conjugated peanut (*Arachis hypogea*) agglutinin (FITC-PNA, EY laboratories inc., San Mateo, CA), E_2_, ZEA, α-ZOL and β-ZOL (all Sigma, St Louis, MO).

### Semen collection and preparation

During the breeding season (March to June 2009), semen was collected repeatedly from five adult stallions. During the experimental period, stallions were housed in straw-bedded boxes and were managed and used in accordance with local health and welfare regulations under strict uniform conditions to ensure that variability in repeated experiments (replicates = 3) were minimally affected by external factors. The use of in-field collected equine semen would not have allowed the present studies because of the potentially large differences in management, feeding and housing. Semen was collected using a Hannover model artificial vagina and immediately filtered though sterile gauze to remove the gel fraction and any debris. The gel-free semen volume was measured using a graduated cylinder, and the initial percentage of motile sperm was estimated by microscopic evaluation; a total motility of ≥ 60% was considered acceptable for subsequent use. Semen samples were washed in HEPES-buffered Tyrode's solution (HBT; 120 mM NaCl, 21.7 mM lactate, 20 mM Hepes, 5 mM glucose, 3.1 mM KCl, 2.0 mM CaCl_2_, 1.0 mM pyruvate, 0.4 mM MgSO_4_, 0.3 mM NaH_2_PO_4 _and 100 μg/ml kanamycin; 300 mOsm/kg, pH 7.4) by centrifugation three times for 5 minutes at 600 × g to remove seminal plasma. After each washing step, the sperm pellet was re-suspended in HBT at a concentration of approximately 100 × 10^6 ^spermatozoa/ml.

Prior to incubation, HBT medium was supplemented with 5 mg/ml of BSA and 15 mM bicarbonate (HBT+BIC) and brought into equilibrium with 5% CO_2 _in humidified air (i.e. capacitating conditions).

### Incubation of spermatozoa with 17β-estradiol and mycotoxins

Stock solutions were prepared for E_2_, ZEA, α-ZOL and β-ZOL by dissolving them in dimethyl sulphoxide (DMSO) at a concentration of 100 mM. Subsequently, nine concentrations of E_2 _and each mycotoxin, ranging from 20 × 10^-5 ^μM to 20 × 10^3 ^μM, were prepared by serial 1:10 dilution of stock solution with DMSO.

The maximum amount of DMSO that did not negatively affect viability, motility or acrosome integrity of stallion sperm was achieved at a 1:200 dilution (reagent in incubation medium). Subsequently, in all experiments, a total of 5 μl of the various concentrations of E_2 _or ZEA derivatives was added to 995 μl of sperm suspended in HBT+BIC at a final concentration of 20 × 10^6 ^spermatozoa/ml such that the final concentration of tested compounds ranged from 1 pM to 0.1 mM. This wide range of concentrations tested encompassed those reported for ZEA, its derivatives and E_2 _in biological fluids, but also included concentrations approximately 10 times higher and 10 times lower than those expected *in vivo*. Control observations were performed in the absence of the test compounds and analyzed at time 0 and after 2 hours of incubation at 38.5°C in an atmosphere of 5% CO_2_-in-air. Further controls were performed by adding 5 μl DMSO to the sperm suspension and tested after 2 hour incubation. Experimental measurements were performed at the end of the 2 hour incubation.

### Sperm motility analysis

Computer-assisted sperm motility analysis (CASA) was performed using a Sperm Vision^® ^(Minitüb GmbH, Tiefenbach, Germany) system equipped with a heated stage. Seven motility parameters were recorded for subsequent analysis; percentages of motile sperm (total and progressively motile), the average path velocity (VAP; expressed in μm/sec), the straight-line velocity (VSL; μm/sec), the curvilinear velocity (VCL; μm/sec), the amplitude of lateral head displacement (ALH; μm) and the linearity (LIN;%). Each evaluation was performed at the same sperm concentration (20 × 10^6 ^spermatozoa/ml) and included analysis of 12 microscope fields (as suggested by the manufacturer) in three ejaculates recovered from each of the five stallions included in the study. For each motility parameter, data were recorded as mean (± standard deviation).

### Acrosome status examination

Acrosome status and sperm viability were examined simultaneously by staining samples with 1 μg/ml FITC-PNA and 1 μM PI, respectively. Sperm samples were incubated in the dark for 10 minutes at 37°C in an atmosphere of 5% CO_2_-in-air. Sperm suspensions were then further diluted with HBT+BIC to a concentration of 2 × 10^6 ^spermatozoa/ml before flow cytometric analysis (FACScan, Becton Dickinson, San Jose, CA), which involved the acquisition of 10,000 events at a flow rate of approximately 200 cells per second, as described by Flesch et al. [33]. The resulting data was analyzed using Cell-Quest software (Becton Dickinson). Samples incubated in the presence of 1 μM calcium ionophore A23187 were used as positive controls for the induction of the AR. The percentages of live, acrosome-reacted and of dead cells were recorded. For each of the five stallions, each compound was tested on one ejaculate over the full range of concentrations.

### Statistical analysis

Data for motility parameters and acrosome status were analyzed using mixed model analyses of variance from the SAS™ package v. 9.1, (SAS Institute, Cary, NC). In the models, the dose-response effects of E_2_, ZEA and its derivatives (α-ZOL and β-ZOL) were considered as fixed effects, whereas the stallion was treated as a random effect. Repeatability of motility parameters was computed from the components of variance obtained by a mixed model procedure on several individuals from which two or more measurements were performed.

## Results

**Figure 1:**
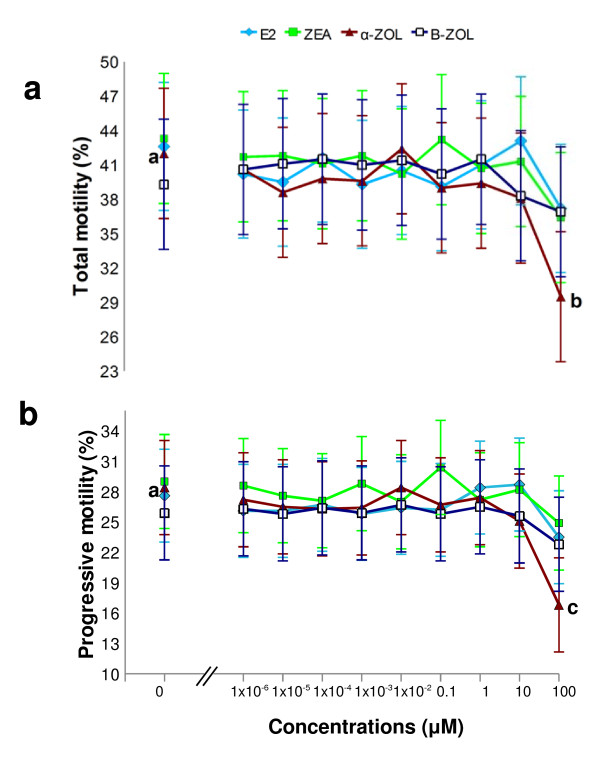
**Dose-response effect of estrogenic mycotoxins and 17β-estradiol on total and progressive motility of stallion sperm**. As shown in panels **a **and **b**, *in vitro *exposure to 0.1 mM α-ZOL significantly depressed total (p < 0.05) and progressive (p = 0.0001) motility, compared to controls ("0 μM"). Zearalenone, β-ZOL and E_2 _did not affect these two motility parameters at any concentration examined. Least Square Means obtained by the mixed model procedure. Different letters indicate values that differ significantly. a *vs *b = p < 0.05; a *vs *c = p = 0.0001; n = 5, replicates = 3.

**Figure 2:**
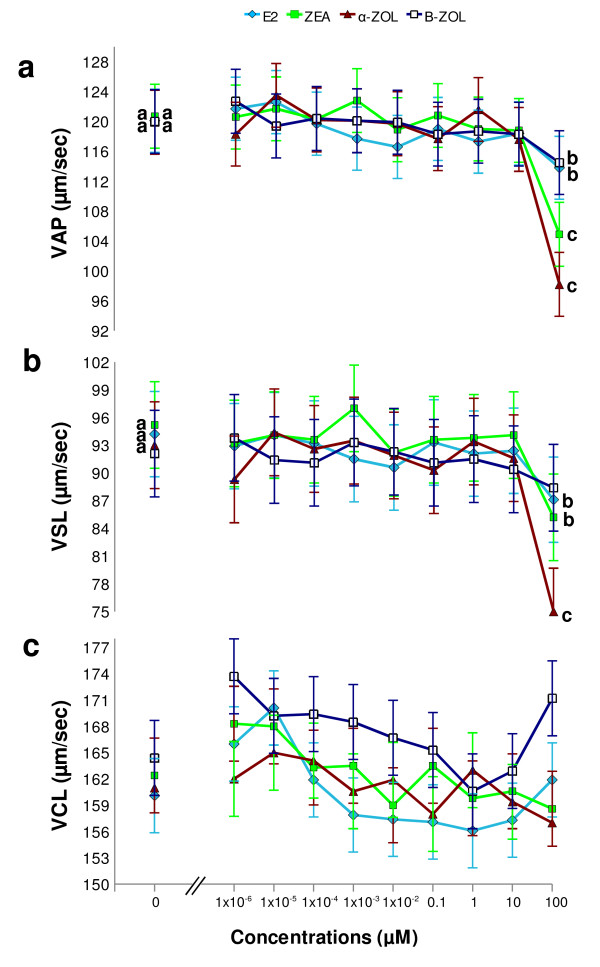
**Dose-response effect of estrogenic mycotoxins and 17β-estradiol on VAP, VSL and VCL of stallion sperm**. As shown in panel **a**, VAP was significantly reduced after *in vitro *exposure to 0.1 mM ZEA (p < 0.0001), α-ZOL ( p < 0.0001), β-ZOL (p < 0.05) and E_2 _(p < 0.05)¸ compared to controls ("0 μM"). Panel **b**: VSL was significantly reduced after *in vitro *exposure to 0.1 mM ZEA (p < 0.05), α-ZOL (p < 0.0001) and E_2 _(p < 0.05), compared to controls ("0 μM"). Panel **c**: Estrogenic mycotoxins and E_2 _did not modify VCL at any concentration examined. Different letters indicate values that differ significantly a *vs *b = p < 0.05; a *vs *c = p < 0.0001; n = 5, replicates = 3.

**Figure 3:**
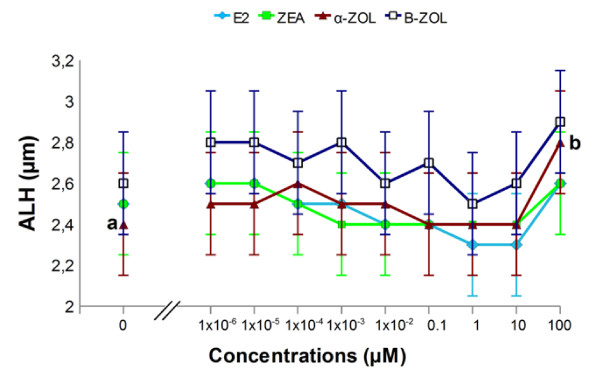
**Dose-response effect of estrogenic mycotoxins and 17β-estradiol on lateral head displacement (ALH) of stallion sperm**. *In vitro *exposure to 0.1 mM α -ZOL significantly increased (p < 0.05) ALH, compared to control ("0 mM"), whereas ZEA, β-ZOL and E_2 _did not modify this parameter at any concentration examined. Different letters indicate values that differ significantly a *vs *b = p < 0.05; n = 5, replicates = 3.

**Figure 4:**
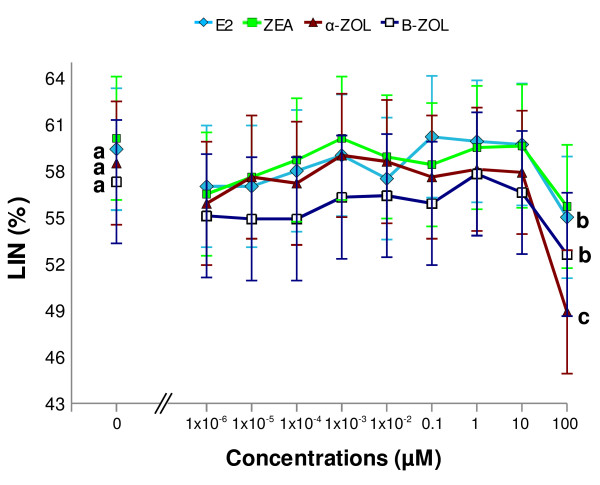
**Dose-response effect of estrogenic mycotoxins and 17β-estradiol on linearity (LIN) of stallion sperm**. *In vitro *exposure to 0.1 mM α-ZOL, β-ZOL and E_2 _significantly reduced LIN (p < 0.0001; p < 0.05 and p < 0.05, respectively), compared to control ("0 μM"), whereas ZEA did not modify this parameter at any examined concentration. Least Square Means obtained by the mixed model procedure. Different letters indicate values that differ significantly a *vs *b = p < 0.05; a *vs *c = p < 0.001; n = 5, replicates = 3.

**Figure 5:**
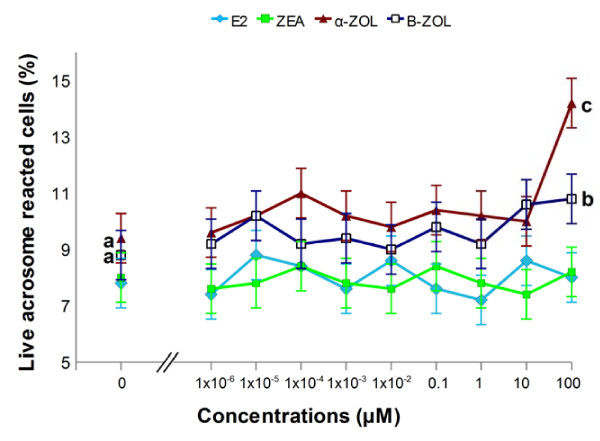
**Dose-response effect of estrogenic mycotoxins and 17β-estradiol on proportion of live acrosome-reacted stallion sperm**. Exposure to α- and β-ZOL at a concentration of 0.1 mM significantly increased the percentage of (live) acrosome reacted sperm (p < 0.0001 and p < 0.05, respectively), when compared to controls ("0 μM"). No effects of ZEA or E_2 _on the percentage of live acrosome reacted cells were observed. Different letters indicate values that differ significantly a *vs *b = p < 0.05; a *vs *c = p < 0.001; n = 5.

In this study, most of the motility parameters and the percentages of sperm undergoing the AR were significantly and dose-dependently influenced by addition of E_2 _or the mycotoxin ZEA and its derivatives, as shown in Table 1. A significant influence of stallion was also evident for all parameters measured (Table 1). By using a mixed model procedure, the dose-response effects of E_2_, ZEA and its derivatives (α-ZOL and β-ZOL) were tested after correction for the stallion effect. Moreover, acceptable (>0.5) repeatability (on the basis of homogeneity of measurements within individual stallions) was found for all parameters, as shown in Table 1. The adverse and positive effects of the four estrogenic substances on sperm motility are detailed in Figures 1, 2, 3 and 4, and those for the induction of the AR in Figure 5; all effects are also described below in the specific results sections.

**Table 1:** The effect of concentration, substance and stallion on sperm parameters (P values) and repeatability values

Effect	Total motility	Progressive motility	VAP	VCL	VSL	ALH	LIN	Acrosome reaction
concentration	0.053	**0.004**	**<0.0001**	**0.007**	**<0.0001**	**<0.0001**	**<0.0001**	**0.006**
substance	0.32	0.08	0.27	**0.0004**	0.051	**<0.0001**	**0.0007**	**<0.0001**
stallion	**<0.0001**	**<0.0001**	**<0.0001**	**<0.0001**	**<0.0001**	**<0.0001**	**<0.0001**	**<0.0001**
*repeatability*	*0.54*	*0.61*	*0.63*	*0.81*	*0.59*	*0.7*	*0.63*	*n.e*.

*n.e*. = not evaluated due to the absence of replicates

n = 5, replicates = 3 for motility parameters; n = 5, no replicates for acrosome reaction

### Dose-dependent effect of *in vitro* exposure to 17β-estradiol, zearalenone and its derivatives on sperm motility

Motility parameters observed in sperm samples exposed *in vitro *to E_2_, ZEA, α-ZOL and β-ZOL are depicted in Figures 1-4. In order to examine whether ZEA and its derivatives induced hyperactivation of the motile sperm (an index of capacitation), four motility parameters, namely VSL, VCL, ALH and LIN were examined as stipulated below. These motility parameters were chosen as they have been shown to change during equine sperm hyperactivation; in this respect, VCL and ALH have been reported to increase, and VSL and LIN to decline [34,35].

#### 17β-Estradiol

*In vitro *exposure to E_2 _did not modify total or progressive motility at any concentration examined (Figures 1a and 1b) when compared to controls (2 hour exposure to DMSO without E_2_). On the other hand, at a concentration of 0.1 mM, E_2 _significantly reduced VAP and VSL (p < 0.05; Figures 2a and 2b), whereas it had no effect on VCL (Figure 2c) or ALH (Figure 3), and caused a reduction in LIN (p < 0.05; Figure 4),

#### Zearalenone

*In vitro *exposure to ZEA did not alter total or progressive motility at any concentration examined (Figures 1a and b). At a concentration of 0.1 mM, ZEA significantly reduced VAP (p < 0.0001; Figure 2a) and VSL (p < 0.05; Figure 2b), whereas it had no effect on VCL (Figure 2c), ALH or LIN at any of the examined concentrations (Figures 3 and 4).

#### α-Zearalenol

*In vitro *exposure to 0.1 mM α-ZOL significantly depressed total (p < 0.05) and progressive (p 7 = 0.0001) motility (Figures 1a and 1b). In addition, exposure to 0.1 mM α-ZOL reduced VAP (p < 0.0001; Figure 2a), VSL (p < 0.0001; Figure 2b) and LIN (p < 0.0001; Figure 4), but did not affect VCL (Figure 2c) and caused an increase in ALH (p < 0.05; Figure 3).

#### β-Zearalenol

*In vitro *exposure to β-ZOL did not affect total or progressive motility at any concentration examined (Figures 1a and b). At 0.1 mM, β-ZOL significantly reduced VAP (p < 0.05; Figure 2a) and LIN (p < 0.05; Figure 4), whereas it had no effect on VSL, VCL (Figures 2b and c) or ALH at any examined concentration.

### Acrosome integrity and sperm viability after *in vitro* exposure to 17β-estradiol and mycotoxins

No effects of E_2 _or ZEA on the percentage of live acrosome reacted sperm (Figure 5) were observed, when compared to controls. However, exposure to α-ZOL or β-ZOL, at a concentration of 0.1 mM, significantly increased the percentage of live acrosome-reacted sperm (p < 0.0001 and p < 0.05, respectively). When cells were exposed to calcium ionophore, as a positive control for the induction of the AR, the percentage of live acrosome-reacted sperm also (p < 0.0001) increased significantly (10.6%), when compared to controls (7.6%). The percentage of dead cells was not affected by exposure to either E_2_, ZEA or its derivatives at any of the examined concentrations (data not shown).

## Discussion

In the present study, we analyzed the effects of mycotoxins and E_2 _on motility (including characteristics of hyperactivated motility) and acrosome integrity in stallion sperm exposed to E_2 _or estrogenic mycotoxins for 2 hours. In general, it is accepted that capacitation and hyperactivation are linked events in the continuum of changes that a sperm must undergo in preparation for fertilization of an oocyte, and previous reports have suggested that stallion sperm require relatively long incubations to induce signs of capacitation [34-38]. For example, McPartlin et al. [35] reported that a 6 hour incubation was needed to observe procaine-induced hyperactivation in stallion sperm. In the present study, a 2 hour incubation was chosen for both motility (CASA) and flow cytometric analysis because, in our experimental conditions, longer incubation times (e.g. 4 hours) significantly reduced sperm viability. Moreover in a previous study [6], we reported deleterious effects of mycotoxins on the chromatin structure stability of stallion sperm after 2 hours of incubation.

The results of the present study indicate that a 2 hour *in vitro *exposure to α-ZOL affected proportions of motile sperm (total and progressive motility) only when the toxin was present at a high concentration (0.1 mM). The effects of α-ZOL on the proportion of motile equine sperm resembled those previously reported for ZEA and α-ZOL on boar sperm [17,18], even though different experimental conditions were used in the earlier studies. In the current study, exposure to E_2 _did not affect the percentages of motile sperm (total or progressive); this corresponds with the findings of Luconi et al. [39] for human sperm. On the other hand, at high concentrations (0.1 mM) both E_2 _and ZEA and its derivatives affected VAP; this suggests that, while the percentages of motile sperm are not affected by estrogenic mycotoxins, the speed at which the sperm move is consistently reduced by exposure to this family of mycotoxins. In particular, a pronounced reduction in VAP was observed after exposure to α-ZOL or ZEA. Again, the findings for the effect of E_2 _are in agreement with those reported by Luconi et al. [40] for human sperm. With respect to the effects on motility parameters, the following rank of activity was apparent: α-ZOL > ZEA > β-ZOL = E_2_. Moreover, at the concentrations examined, α-ZOL was the only ZEA derivative able to reduce the proportion of motile sperm in stallion semen, whereas all the tested mycotoxins influenced the velocity of sperm movement of the remaining motile cells and, in addition, there was a trend for the sperm motility parameters to change in a way suggesting hyperactivation of motility; i.e., reductions in VSL and LIN and a concomitant increase in ALH. More specifically, at the highest tested concentration (0.1 mM), ZEA inhibited VSL, α-ZOL reduced VSL and LIN and increased ALH, and β-ZOL reduced LIN. At the same concentration, E_2 _reduced VSL and LIN. In the only previous report [19] of the effect of ZEA derivatives on sperm motility parameters, β-ZOL and α-ZOL were found to stimulate VCL in boar sperm when introduced at concentrations ranging from 0.2 to 20 μM, after 5 hours of incubation. The current study is the first to examine the effects of E_2 _on sperm motility parameters associated with hyperactivation. The ranking of the effects on hyperactivation-related motility parameters in stallion sperm was α-ZOL > E_2 _> ZEA = β-ZOL.

Neither ZEA nor E_2 _influenced the proportion of live acrosome-reacted sperm during a 2 hour incubation. By contrast, 0.1 mM α-ZOL or β-ZOL stimulated an increase in the percentage of live acrosome-reacted sperm. In a previous study on pig sperm [17], ZEA and α-ZOL at higher concentrations (125 to 250 μM) reduced the proportion of live acrosome-reacted cells and concomitantly increased the proportion of deteriorated (dead) sperm. These conflicting results could relate to differences in the experimental conditions used, such as incubation time and methods of determination. We did not observe the deterioration effect and, besides the likely contribution of the relatively short incubation employed in the current study, we note the advice that detection of the acrosome status of incubated sperm should preferably be performed at the incubation temperature because temperature shock can easily induce membrane damage [41]. In the present study, E_2 _did not influence the percentage of live acrosome-reacted sperm.

Conflicting data on whether, and at what molecular level, estrogens may affect the AR are still not completely resolved. The currently favored theory is that E_2 _inhibits the progesterone- or ATP-induced AR in both human [23,42,43] and bovine [44] sperm. However, studies in the mouse have documented a stimulatory effect of E_2 _on the AR [45,46]. On the other hand, it may be significant that different incubation conditions (medium, time), staining (FITC-PNA or FITC-Pisum sativum agglutinin – PSA) and evaluation techniques (fluorescence microscope or flow cytometry) were used in the various studies. In the current study, a ranking of the magnitude of the effect on the AR of stallion sperm was Beta-zearalenol > β-ZOL > E_2 _= ZEA.

In the present study, a 2 hour incubations in the presence of ZEA, its derivatives or E_2 _were sufficient to induce changes in equine sperm motility, hyperactivated motility, and in the percentage of sperm that had acrosome-reacted. The most potent compound was α-ZOL, which modified sperm motility (total and progressive motility), sperm velocity (VAP), most of parameters related to hyperactive motility (VSL, LIN and ALH) and induced the AR to a larger extent than any of the other estrogenic compounds studied. Beta-zearalenol affected only the percentage of acrosome-reacted sperm. As a result of the contrasting effects of related chemicals, there must be suspicions that, in the stallion, the pathways that regulate capacitation and hyperactivation are separate and independent, as previously reported in other species [47-49].

Apparently, the mycotoxin ZEA influenced hyperactivation-associated parameters to a lesser extent than E_2_, whereas both ZOL derivatives induced premature acrosome reactions and thereby adversely affected the fertilizing capacity of stallion sperm. The α isomer of ZOL still possessed the apparently estrogenic capacity to induce hyperactivated motility, whereas the β stereo-isomer had lost this property.

Importantly, the current study showed that neither ZEA, its derivatives nor E_2 _affected sperm cell deterioration during the 2 hour incubation. Previous studies reported a significant decrease in the percentage of viable boar sperm after *in vitro *exposure to ZEA, α-ZOL and β-ZOL [17,19]; however, this occured at higher concentrations of mycotoxin [17] and at longer incubation times than in the present study [19].

## Conclusions

In the experimental conditions used, α-ZOL was the only ZEA derivative able to decrease the percentage of motile sperm cells, induce changes in motility characteristics of the remaining motile sperm consistent with hyperactivation, and induce the AR in stallion sperm. Zearalenone and β-ZOL induced only some of the motility parameters characteristic of hyperactivation and, of the two, only β-ZOL induced the AR. None of the mycotoxins behaved identically to E_2_, which induced hyperactivation-like changes in sperm motility without inducing the AR. These results differ to those reported in other species, probably in large part because of the different experimental conditions employed. It is, however, of interest that ZEA and E_2 _had similar effects restricted to stimulating hyperactivation of stallion sperm, while both ZOL stereo-isomers had additional effects at the level of AR induction. The effect on hyperactivation of sperm was also present for α-ZOL (and even more potent) but was lost for the stereo-isomer β-ZOL. This suggests that metabolism of ZEA converts an initially estrogenic compound into (a) a more active component with respect to its capacity to induce hyperactivation of equine sperm and (b) a compound capable of additional activation of the AR. Both effects are presumably elicited at a premature (i.e. before sperm-oocyte interaction in the oviduct) stage of reproductive physiology and are therefore likely to inhibit fertilizing potential of affected sperm.

## Competing interests

The authors declare that they have no competing interests.

## Authors' contributions

FM, MED and BC conceived the study. TAES and BG designed the study. FM provided the background on mycotoxins. TAES and BMG coordinated flow cytometer and CASA settings. AF performed flow cytometry trials, under the guide of FM, BG and ES. AF performed CASA analysis, under the guide of TAES and ES. FP and AF performed statistical analysis. AF, FM, MED, TAES, BG and FP prepared the graphs and tables. FM, MED, TAES and AF discussed results interpretation and wrote the manuscript. BMG and BC participated in experiment supervision and in critical reading of the manuscript. All the authors read and approved the final manuscript.
